# Stably high azithromycin resistance and decreasing ceftriaxone susceptibility in *Neisseria gonorrhoeae* in 25 European countries, 2016

**DOI:** 10.1186/s12879-018-3528-4

**Published:** 2018-12-03

**Authors:** Michaela J. Day, Gianfranco Spiteri, Susanne Jacobsson, Neil Woodford, Andrew J. Amato-Gauci, Michelle J. Cole, Magnus Unemo, Euro-GASP network, Alexander Indra, Steliana Huhlescu, Wim Vanden Berghe, Tania Crucitti, Blaženka Hunjak, Tatjana Nemeth Blažić, Jan Kubele, Hana Zákoucká, Helena Žemličková, Lene Berthelsen, Susan Cowan, Steen Hoffmann, Jevgenia Epstein, Jelena Viktorova, Ndeindo Ndeikoundam, Agathe Goubard, Beatrice Bercot, Peter Kohl, Susanne Buder, Viviane Bremer, Klaus Jansen, Eva Tzelepi, Vasileia Konte, Eszter Balla, Mária Dudás, Guðrún Sigmundsdóttir, Guðrún Svanborg Hauksdóttir, Derval Igoe, Brendan Crowley, Barbara Suligoi, Paola Stefanelli, Gatis Pakarna, Violeta Mavcutko, Paul Reichert, Patrick Hoffmann, Christopher Barbara, Francesca Vella, Jackie Maistre Melillo, Alje Van Dam, Birgit Van Benthem, Ineke Linde, Hilde Kløvstad, Martin Steinbakk, Sławomir Majewski, Jacinta Azevedo, Maria-José Borrego, Peter Pavlik, Peter Truska, Irena Klavs, Samo Jeverica, Julio Vazquez, Asuncion Diaz, Raquel Abad, Inga Velicko, Magnus Unemo, Gwenda Hughes, Kate Templeton, Neil Irvine

**Affiliations:** 1 National Infection Service, Public Health England, London, UK; 2 European Centre for Disease Prevention and Control, Stockholm, Sweden; 3 WHO Collaborating Centre for Gonorrhea and other STIs, Örebro University, Örebro, Sweden

**Keywords:** Gonorrhoea, Treatment, Antimicrobial resistance, Ceftriaxone, Azithromycin, Surveillance, European gonococcal antimicrobial surveillance Programme (Euro-GASP), Europe, European Union (EU), European Economic Area (EEA)

## Abstract

**Background:**

The European Gonococcal Antimicrobial Surveillance Programme (Euro-GASP) performs annual sentinel surveillance of *Neisseria gonorrhoeae* susceptibility to therapeutically relevant antimicrobials across the European Union/European Economic Area (EU/EEA). We present the Euro-GASP results from 2016 (25 countries), linked to patient epidemiological data, and compared with data from previous years.

**Methods:**

Agar dilution and minimum inhibitory concentration (MIC) gradient strip methodologies were used to determine the antimicrobial susceptibility (using EUCAST breakpoints) of 2660 *N. gonorrhoeae* isolates from 25 countries across the EU/EEA. Significance of differences compared with Euro-GASP results in previous years was analysed using Z-tests.

**Results:**

No isolates with resistance to ceftriaxone (MIC > 0.125 mg/L) were detected in 2016 (one in 2015). However, the proportion of isolates with decreased susceptibility to ceftriaxone (MICs from 0.03 mg/L to 0.125 mg/L) increased significantly (*p* = 0.01) from 2015 to 2016. There were 14 (0.5%) isolates with ceftriaxone MICs 0.125 mg/L (on the resistance breakpoint), of which one isolate was resistant to azithromycin and four showed intermediate susceptibility to azithromycin. Cefixime resistance was detected in 2.1% of isolates in 2016 compared with 1.7% in 2015 (*p* = 0.26) and azithromycin resistance in 7.5% in 2016 compared with 7.1% in 2015 (*p* = 0.74). Seven (0.3%) isolates from five countries displayed high-level azithromycin resistance (MIC≥256 mg/L) in 2016 compared with five (0.2%) isolates in 2015. Resistance rate to ciprofloxacin was 46.5% compared with 49.4% in 2015 (*p* = 0.06). No isolates were resistant to spectinomycin and the MICs of gentamicin remained stable compared with previous years.

**Conclusions:**

Overall AMR rates in gonococci in EU/EEA remained stable from 2015 to 2016. However, the ceftriaxone MIC distribution shifted away from the most susceptible (≤0.016 mg/L) and the proportion of isolates with decreased susceptibility to ceftriaxone increased significantly. This development is of concern as current European gonorrhoea management guideline recommends ceftriaxone 500 mg plus azithromycin 2 g as first-line therapy. With azithromycin resistance at 7.5%, the increasing ceftriaxone MICs might soon threaten the effectiveness of this therapeutic regimen and requires close monitoring.

## Background

The treatment and control of gonorrhoea during the last decade has been under serious threat due to the emergence and spread of antimicrobial resistance (AMR) in *Neisseria gonorrhoeae.* Ceftriaxone is the last remaining option for effective empiric first-line antimicrobial monotherapy. The current European gonorrhoea management guideline recommends combination treatment with ceftriaxone plus azithromycin in an attempt to mitigate the development and/or spread of resistance to these antimicrobials [1]. Susceptibility to these antimicrobials has decreased internationally in the past [2, 3], and in 2018, the first gonococcal strain with ceftriaxone resistance combined with high-level resistance to azithromycin was reported from England [4], which was followed by two similar cases in Australia [5]. Therefore, surveillance of gonococcal susceptibility, including monitoring current and emerging trends in AMR, is essential in order to ensure effective patient management [6, 7].

Since 2009, the European Gonococcal Antimicrobial Surveillance Programme (Euro-GASP), coordinated by the European Centre for Disease Prevention and Control (ECDC) and supported by a European network of microbiologists and epidemiologists, has strengthened the surveillance of gonococcal antimicrobial susceptibility in the European Union (EU)/European economic area (EEA), in order to provide quality-assured data to inform gonorrhoea treatment guidelines. Euro-GASP has identified decreasing susceptibility to extended-spectrum cephalosporins in the past and treatment failures have been documented [6–9], prompting the creation and subsequent implementation of a European response plan to control and manage the threat of multidrug-resistant *N. gonorrhoeae* in Europe [7].

This study presents the results from the 2016 European gonococcal antimicrobial susceptibility sentinel surveillance, in conjunction with patients’ epidemiological data, and compares the results to data from previous years.

## Methods

### European gonococcal antimicrobial surveillance Programme (euro-GASP)

Participating laboratories from 25 EU/EEA countries (Table 1) collected *N. gonorrhoeae* isolates from consecutive patients (one isolate per patient) from September to November 2016 unless sufficient numbers could not be achieved during this time frame, in which case isolates were collected throughout the year. The United Kingdom (UK) collected isolates from July to September 2016 (to coincide with the collection in the national Gonococcal Resistance to Antimicrobials Surveillance Programme (GRASP) [10]) and isolates were selected from this collection to give full UK geographical representation. The isolate collection period was the same as 2015. Full details of the Euro-GASP *N. gonorrhoeae* isolate collection and selection criteria can be found in the Euro-GASP protocol [11]. Antimicrobial susceptibility testing was performed using MIC gradient strip tests or agar dilution methods (determination of minimum inhibitory concentration (MIC) or breakpoint technique) for ceftriaxone, cefixime, azithromycin, ciprofloxacin, spectinomycin and gentamicin as previously described [11]. Since 2014, spectinomycin and gentamicin are only tested every 3 years as they are not in routine use for treatment, susceptibility has appeared to be stably high, and spectinomycin is difficult to acquire. Isolates from eight (32%) countries (Table 1) were tested centrally at Public Health England or Örebro University Hospital, Sweden with the remaining 17 (68%) countries performing antimicrobial susceptibility testing in their own laboratories (these laboratories successfully fulfilled established quality criteria prior to commencing their own testing). All Euro-GASP laboratories participated in an annual external quality assessment (EQA) programme [12] to ensure comparability of antimicrobial susceptibility data. The antimicrobial susceptibility data were interpreted into susceptibility, intermediate susceptibility and resistance using EUCAST resistance breakpoints: cefixime/ceftriaxone resistance MICs > 0.125 mg/L, azithromycin resistance MIC > 0.5 mg/L, ciprofloxacin resistance MIC > 0.06 mg/L, and spectinomycin resistance MIC > 64 mg/L [13]. In addition to antimicrobial susceptibility data, patient epidemiological data were collected and categorised; age (< or ≥ 25 years); gender and sexual orientation (men who have sex with men (MSM), male heterosexual or female); anatomical site of collection (urogenital, rectal, pharyngeal, other); previous gonorrhoea (yes/no) and concurrent *Chlamydia trachomatis* infection (yes/no). The overall coverage of Euro-GASP was estimated by comparing the number of isolates tested in Euro-GASP to the number of cases reported as part of the enhanced epidemiological surveillance of STI in 2016 [14].

**Table 1:**
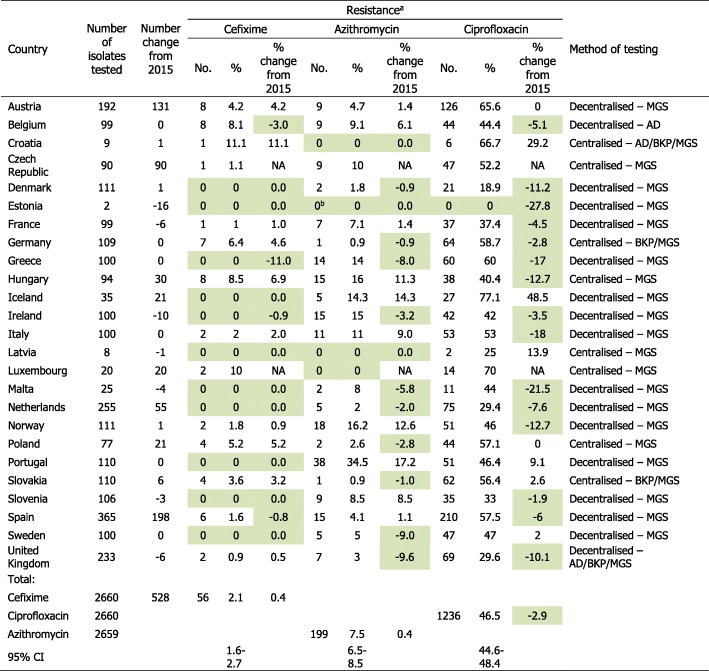
Resistance to cefixime, azithromycin and ciprofloxacin by country, Euro-GASP (25 countries), 2016

*Euro-GASP* European Gonococcal Antimicrobial Surveillance Programme, *No.* Number, MGS MIC gradient strip test (mostly Etest strips used), *AD* agar dilution method to determine the MIC of an antimicrobial, *BKP* Breakpoint agar dilution method, *CI* confidence interval of the mean %, *NA* not applicable (countries not included in Euro-GASP in 2015)

^a^Columns shaded in green indicate a reduction in the percentage of resistant isolates from 2015 to 2016 or a continuation of zero resistance

^b^One isolate tested

Note: Belgium, Germany, the Netherlands and Sweden included isolates collected from September to November, Denmark and Norway from August to November, Ireland and Italy from August to December, Poland from July to December, the United Kingdom from July to December, and the remaining countries collected throughout the year

### Statistical analysis

Statistical analysis was performed using Stata v13.1 (StataCorp LP, Texas, USA). The Z-test was used to determine differences between AMR and epidemiological data collected in 2016 versus 2015 and a Mann-Whitney test was used to test whether the differences in age distribution were statistically significant. Where datasets contained sufficient numbers, associations between patient epidemiological data and AMR were assessed using odds ratios (OR) and 95% confidence intervals (CI); the Pearson’s χ^2^ test was used to measure if these odds ratios differed significantly from 1. For small cell numbers, Fisher’s exact test was performed. Statistical significance for all tests was assumed when *p* < 0.05.

## Results

During 2016, a total of 2660 isolates were tested in Euro-GASP, which represents an increase of 526 isolates (24.6%) compared with 2015. The number of isolates tested from each country varied from two (Estonia) to 365 (Spain) (Table 1). The overall coverage of Euro-GASP was 4% and ranged from 1% to over 100% (indicating incomplete epidemiological surveillance in one country).

The epidemiological characteristics of all patients (2015 and 2016) is summarised in Table 2. Most isolates (85.1%) were from male patients; an increase compared with 2015 (81.8%, *p* < 0.01). Patient ages ranged from < 1 year to 93 years with a median age of 30 years and 27.5% of patients aged < 25 years. Males (median age 30 years) were statistically older (Mann-Whitney *p* < 0.0001) than females (median age 24 years) with the highest and lowest percentage of < 25-year-olds in the female (51.5%) and MSM patient groups (20.1%), respectively. The anatomical site of collection was mainly urogenital (75.5%), followed by rectal (14.2%) and pharyngeal (6.4%). For cases where information was available (31.9%, 849/2660), 17.2% had a previous gonorrhoea infection (stable from 2015; 17.5%) and 23.9% had a concurrent *C. trachomatis* infection, which is an increase from 2015 (19.0%, *p* = 0.01, Z-test). Among cases with known sexual orientation and gender (64.8%, 1723/2660), 59.6% of the *N. gonorrhoeae* infections were reported as heterosexually acquired (38.5% females and 61.5% males) and 40.4% were from MSM.

**Table 2:** Patient characteristics 2015–2016

	2015	2016
	N (%)	N (%)
Total number of isolates	2134	2660
Gender		
Male	1736 (81.8)^ǂǂ^	2256 (85.1)^ ^ǂǂ^
Female	385 (18.2)^ǂǂ^	395 (14.9) ^ǂǂ^
Unknown	13	9
Age (years)		
< 25	617 (29.5)	720 (27.5)
≥25	1476 (70.5)	1902 (72.5)
Unknown	41	38
Sexual orientation & gender		
Females	385 (26.4)^ǂǂ^	395 (22.9) ^ǂǂ^
Heterosexual males	419 (28.7)^ǂǂ^	632 (36.7) ^ǂǂ^
Men who have sex with men	657 (45.0)	696 (40.4)^ ^ǂǂ^
Unknown	673	937
Site of infection		
Genital	1517 (72.9)^ǂǂ^	1943 (75.5)
Pharyngeal	180 (8.7)	165 (6.4) ^ǂǂ^
Anorectal	280 (13.5)^ǂǂ^	366 (14.2)
Other	103 (5.0)^ǂǂ^	100 (3.9)
Unknown	54	86
Previous gonorrhoea		
Yes	157 (17.5)	171 (17.2)
No	739 (82.5)	824 (82.8)
Unknown	1238	1665
Concurrent STI		
Concurrent chlamydia infection	153^††^ (19.0)	203 (23.9)˜ ^ǂǂ^
Concurrent other STI (not HIV)	48^††^ (6.0)	53 (6.2) ^††^
No concurrent STI	605 (75.1)	593 (69.9) ^ǂǂ^
Unknown	1328	1811
HIV status		
Positive	132 (15.3)^ǂǂ^	156 (15.9)
Negative	733 (84.7)^ǂǂ^	823 (84.1)
Unknown	1269	1681

Percentages calculated from known values

^††^Includes four individuals with two concurrent STIs

^ǂǂ^Significant difference compared to previous year (*p* < 0.05)

^Includes one individual of unknown gender, but with mode of transmission reported as MSM

˜ Includes nine individuals with chlamydia and an additionally diagnosed STI

**Fig. 1:**
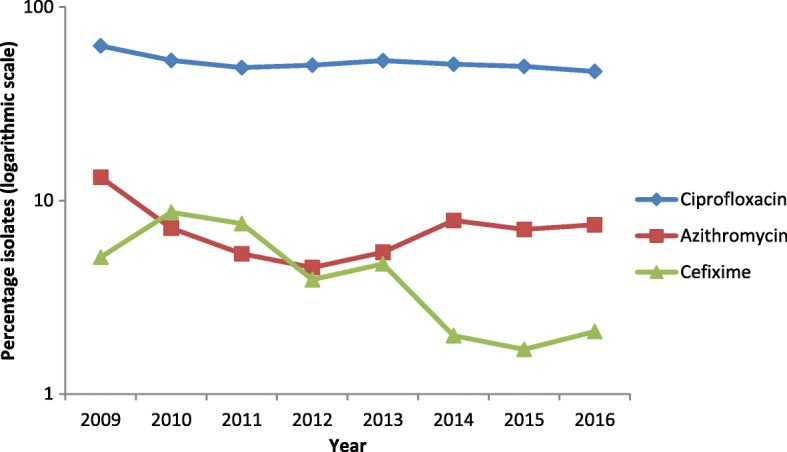
Trends in resistant *Neisseria gonorrhoeae* isolates by antimicrobial and year, Euro-GASP, 2009–2016. Percentage of resistant isolates on logarithmic scale on y-axis. Number of ceftriaxone resistant isolates; 2009 and 2010 *n* = 0, 2011 *n* = 10, 2012 *n* = 3, 2013 *n* = 7, 2014 *n* = 5, 2015 *n* = 1, and 2016 *n* = 0

No isolates displayed ceftriaxone resistance (MIC> 0.125 mg/L) in 2016 compared with one (0.05%) in 2015 (isolated in Greece), five (0.23%) in 2014 and seven (0.35%) in 2013. Fourteen isolates (0.5%) had a ceftriaxone MIC of 0.125 mg/L (on the resistance breakpoint), of which one (7.1%) was resistant to azithromycin (isolate collected in Norway) and four (28.6%; Spain, *n* = 2; Greece, *n* = 1; and Slovakia, n = 1) had an intermediate susceptibility to azithromycin (MIC = 0.5 mg/L). The proportion of gonococcal isolates which were more susceptible to ceftriaxone (MIC≤0.016 mg/L) decreased in 2016 (81.7%) when compared with 2015 (84.2% *p* = 0.03, Z-test). In addition, the proportion of isolates with decreased susceptibility to ceftriaxone (MICs from 0.032 mg/L to 0.125 mg/L) increased to 17.7% in 2016 from 15.0% in 2015 (*p* = 0.01, Z-test) (Fig. 1).

**Fig. 2:**
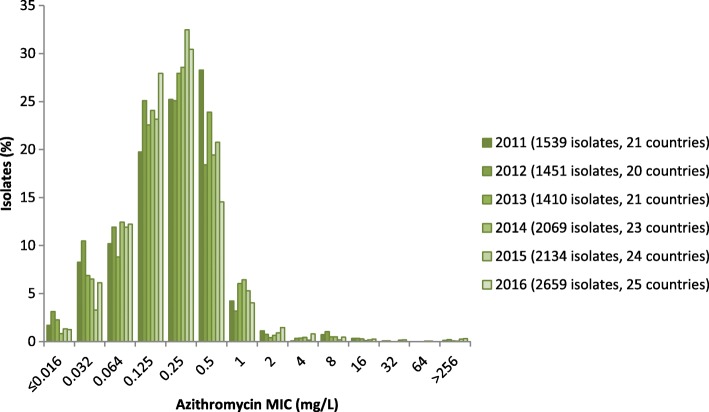
Azithromycin MIC distribution in Euro-GASP, 2011–2016

Azithromycin resistance remained stable: 7.5% (199/2659) in 2016 compared with 7.1% (152/2134) in 2015 (*p* = 0.74, Z-test). The highest azithromycin resistance levels were detected in Portugal (34.5%), Norway (16.2%) and Iceland (14.3%), which differs from 2015 when Greece (22.0%) and Ireland (18.2%) documented the highest levels [11, 15]. Seven (0.3%) isolates displayed high-level resistance to azithromycin (MIC≥256 mg/L), which was the highest number of high-level resistant isolates recorded in Euro-GASP surveillance (2011 *n* = 2, 2012 *n* = 3, 2013 *n* = 1, 2014 n = 1, 2015 *n* = 5) (Fig. 2). These seven isolates were obtained in Iceland (n = 2), Italy (n = 2), Czech Republic, Ireland, and the UK (one isolate each) and were susceptible to all the other antimicrobials tested except for the Czech isolate that displayed ciprofloxacin resistance. The MIC distribution for azithromycin in 2016 was similar to previous years (Fig. 2); the majority of resistant isolates had an MIC just above the resistance breakpoint (0.5 mg/L) and the modal MIC remained at the same level (0.25 mg/L) as in 2015. Fourteen (0.5%) azithromycin resistant isolates were also resistant to cefixime and these isolates were from Hungary (*n* = 8), Belgium (*n* = 3), Italy (*n* = 1), Norway (n = 1), and Poland (n = 1). Eighty (3.0%) azithromycin resistant isolates were also resistant to ciprofloxacin. In 2016, as in 2015 [11], azithromycin resistance was highest among heterosexual males (7.6%) followed by MSM (5.6%) and lowest in females (5.3%).

**Fig. 3:**
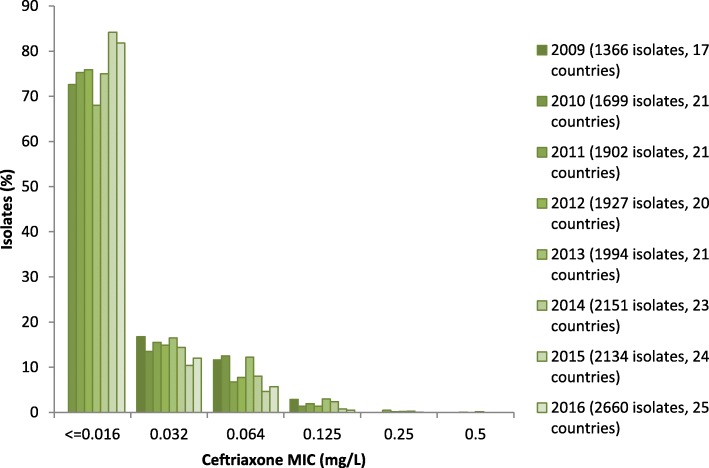
Ceftriaxone MIC distribution in Euro-GASP, 2009–2016

Cefixime resistance remained stable at 2.1% (56/2660) in 2016 compared with 1.7%, (36/2132) (*p* = 0.26, Z-test) in 2015 and remained lower than observed in 2010–2013 (4.7–8.7%) (Table 1, Fig. 3). The increased use of MIC gradient strip tests, which can sometimes read one MIC doubling dilution lower than agar dilution for cefixime [16], may also have had a limited effect on the decrease in cefixime resistance post-2013. The highest cefixime resistance levels were detected in Croatia (11.1%), Luxembourg (10.0%) and Hungary (8.5%), which differs from 2015 when Greece (11.0%) and Belgium (11.1%) had the highest levels [11, 15]. No isolates with a cefixime MIC of ≥0.5 mg/L were detected in 2016, which is a decrease from seven (0.3%) isolates in 2015, three (0.9%) isolates in 2014, 19 (0.2%) isolates in 2013, three (1.0%) isolates in 2012, and 17 (0.1%) isolates in 2011, [15]. The proportion of most susceptible isolates (MIC≤0.016 mg/L) remained stable at 74.3% in 2016 compared with 75.0% in 2015 (*p* = 0.7, Z-test). Cefixime resistance according to sexual orientation and gender was stable in 2016 compared with 2015; highest in heterosexual males (2.2%), followed by females (2% *p* < 0.01, OR = 0.91, CI = 0.38–2.2, Pearson’s χ2 test), and lowest in MSM (0%).

Ciprofloxacin resistance remained stable at 46.5% (1236/2660) compared with 49.4% (1054/2133) in 2015 (*p* = 0.06, Z-test). Resistance was significantly associated with heterosexual males (51.6%, *p* < 0.01, OR = 1.68, CI = 1.35–2.09, Pearson’s χ2 test) and lowest in MSM (38.8%) with a decrease in resistant isolates from both heterosexual males (59.7% in 2015, *p* = 0.01, Z-test) and MSM (44.1% in 2015 *p* = 0.05, Z-test) from 2015 [11, 15] to 2016 with a reciprocal increase in females from 41.8 to 43.3% (*p* = 0.69, Z-test). There was an association between ciprofloxacin resistance and previous gonorrhoea infection (OR = 1.46, CI = 1.05–2.04, *p* = 0.02), which remained in the multivariable analysis (OR = 1.44, CI = 1.02–2.05, *p* = 0.04).

Resistance to azithromycin, cefixime and ciprofloxacin continued to be less common in anorectal isolates (4.6%; 0%; 36.1% respectively) than in urogenital (7.7%; 2.4%; 48.4%) or pharyngeal (7.9%; 0.6%; 38.8%) isolates.

As in previous testing years, there was no spectinomycin resistance detected amongst the 2018 isolates tested across 20 countries. Currently, there are no breakpoints for gentamicin but the MICs continued to be low across all 15 countries that tested 1507 isolates for gentamicin susceptibility in 2016. The MIC ranged from 0.5 mg/L to 16 mg/L with a modal MIC of 4 mg/L which is lower than when last tested in 2013 (modal MIC = 8 mg/L) [17]. This is likely due to a change in methodology with an increased use of Etests in 2016 (83.4% tested by Etest in 2016, 43.6% in 2013) which frequently read one dilution lower than agar dilution for gentamicin [18].

## Discussion

In the EU/EEA, resistance to extended-spectrum cephalosporins remained stable in 2016 with a decrease in the proportion of highly resistant isolates to cefixime (≥0.5 mg/L). However, a reduction in the proportion of isolates highly susceptible to ceftriaxone was observed, which has also been reported from other countries, e.g., China [19] (Changsha 2003–2015 [20], Shandong 2007–2014 [21]) and Argentina (2012–2013) [22] highlighting decreasing ceftriaxone susceptibility in several settings globally.

Azithromycin resistance remained stable at 7.5% in 2016 in EU/EEA, which is comparable to that in the United States (11.1% in 2016 (recalculated using EUCAST breakpoints) [23]), Guangzhou, China (9.1% 2009–2013 [24]) and New Zealand (10.8% 2014–2015 [25]), but higher than that observed in Australia (2.6% in 2015 [26]) and Canada (4.7% in 2015 [27]). Notably, different azithromycin resistance breakpoint were used in Canada (MIC≥2 mg/L [27]) and the United States (MIC≥2 mg/L [23]), which illustrates the difficulties of comparing gonococcal AMR data from different regions or countries and the importance of reporting MIC distributions. Many isolates show MICs close to the EUCAST azithromycin breakpoint which causes resistance rates to fluctuate widely over years which can over emphasise minor shifts in MICs. The induction and/or selection of azithromycin resistance in *N. gonorrhoeae* by the use of dual therapy for gonorrhea is likely limited [28]. Accordingly, the resistance to azithromycin is likely mostly a bystander effect as a consequence of the widespread use of azithromycin monotherapy to treat respiratory tract infections, *C. trachomatis* infections, and/or male non-gonococcal urethritis, which may explain the higher level of azithromycin resistance observed in males in EU/EEA. Increases in *N. gonorrhoeae* isolates with decreased susceptibility to azithromycin from MSM have been observed in Seattle, USA between 2012 and 2016 [29] and more widely across the USA 2013–2014 [30]. Fourteen (7.0%) of the 199 azithromycin resistant isolates in 2016 in EU/EEA were also resistant to cefixime, which is a worrying development that highlights the importance of conserving the efficacy of ceftriaxone as treatment options are becoming increasingly restricted.

Among MSM in EU/EEA, cefixime resistance has remained low and fairly steady since 2013 and MSM remain the risk group with the lowest level of cefixime resistance (0% in 2016). There was a decrease in ciprofloxacin resistance from 2015 (44.1%) to 2016 (38.8%) further supporting the previous finding that MSM currently have a lower risk of harbouring resistant isolates [31]. This is in contrast to the findings in the USA where the percentage of isolates resistant to ciprofloxacin were greater in MSM than in heterosexuals [30].

Even though the number of countries participating (currently 25 (81%) of the 31 EU/EEA countries) and number of isolates in Euro-GASP increased in 2016, the percentage of isolates tested in Euro-GASP compared with the number of gonorrhoea cases reported has decreased from 6% in 2009 to 4% in 2016, which is mainly a result of the increase in the overall number of gonorrhoea cases diagnosed in the EU/EEA [14]. A detailed review of the number and characteristics of the gonococcal isolates submitted to Euro-GASP and their corresponding gonorrhoea patient data is required in order to ensure that Euro-GASP data remain representative of the European *N. gonorrhoeae* population. One of the aims of Euro-GASP is to develop capacity for culture and antimicrobial susceptibility testing across countries allowing for increased numbers of isolates to be included in the study. Additional limitations of Euro-GASP include, as in most national and particularly international GASPs, lower number of isolates from females (15% of isolates in 2016), suboptimal level of reporting of some key epidemiological data such as sexual orientation (sexual orientation and gender were reported for 65% of Euro-GASP cases in 2016), as well as low number and likely suboptimal representativeness of isolates from some countries (e.g. Croatia, Estonia and Latvia) and the need to include six EU/EEA countries. Consequently, it remains crucial to improve the representativeness of Euro-GASP data, which is also part of the ongoing Euro-GASP work programme.

## Conclusions

Even though Euro-GASP detected no resistance to ceftriaxone, stably low resistance to cefixime (2.1%) and stable azithromycin resistance (7.5%) in 2016, the decreasing ceftriaxone susceptibility and the relatively high azithromycin resistance, including isolates with very high azithromycin MICs (≥256 mg/L), is of major concern. The European response plan to control the threat of multidrug-resistant *N. gonorrhoeae* in Europe [7] should continue to be implemented to increase the quality-assured gonococcal AMR surveillance and to support timely identification and reporting of treatment failures and ensure that gonorrhoea remains a treatable infection. Euro-GASP continues to fulfil the objectives of the response plan which include strengthening the surveillance of gonococcal antimicrobial susceptibility by increasing the number of participating countries and isolates and improving representativeness of the programme, advocating the use of recommended dual therapy (ceftriaxone 500 mg and azithromycin 2 g) to treat gonorrhoea [1], which appears highly effective, and ensuring that all Euro-GASP laboratories continue to participate in the EQA programme.

## Abbreviations

AD: Agar dilution method
AMR: Antimicrobial resistance
BKP: Breakpoint agar dilution method
CI: Confidence interval
ECDC: European Centre for Disease Prevention and Control
EEA: European Economic Area
EQA: External quality assessment
EU: European Union
Euro-GASP: European Gonococcal Antimicrobial Surveillance Programme
GRASP: Gonococcal Resistance to Antimicrobials Surveillance Programme
MGS: MIC gradient strip test
MIC: Minimum inhibitory concentration
MSM: Men who have sex with men
No.: Number
OR: Odds ratio
PHE: Public Health England
STI: Sexually transmitted infection
TESSy: The European Surveillance System
UK: United Kingdom
WHO: World Health Organization
